# Porcine Parvovirus in China: Recent Advances, Epidemiology, and Vaccine Strategies

**DOI:** 10.3390/v17091262

**Published:** 2025-09-18

**Authors:** Yunchao Liu, Yumei Chen, Yanli Shang, Xiuli Deng, Huifang Hao

**Affiliations:** 1 Institute for Animal Health, Key Laboratory of Animal Immunology, Henan Academy of Agricultural Sciences, Zhengzhou 450002, China; 2 School of Life Sciences, Zhengzhou University, Zhengzhou 450001, China

**Keywords:** porcine parvovirus, genetic evolution, virus-like particles, reproductive failure, diagnostic, vaccine efficacy

## Abstract

Porcine parvovirus (PPV), a non-envelope single-stranded DNA virus, causes severe reproductive disorders in swine worldwide, characterized by fetal mortality, mummification, and reduced boar fertility. As a highly prevalent pathogen in Chinese swine herds, PPV imposes substantial economic burdens on intensive pig production systems. This review systematically synthesizes recent advances in PPV virology, focusing on genomic evolution of emerging strains (PPV1–PPV8), epidemiological dynamics of emerging strains, molecular pathogenesis, and novel diagnostic tools. Furthermore, we critically evaluate current vaccine strategies, highlighting their limitations in cross-protective efficacy and viral shedding control. By integrating multi-omics insights with immunological profiling, this work delineates actionable pathways for next-generation vaccine design and proposes a roadmap for rational antigen selection. This review consolidates foundational knowledge and establishes a translational bridge between basic virology and prevention and control of porcine parvovirus, addressing critical gaps in porcine reproductive disease management.

## 1. Introduction

Porcine parvovirus (PPV) is one of the main pathogens causing reproductive disorders in pigs and is widely prevalent worldwide [1]. This virus was first isolated and identified in 1965 from cellular contaminants of classical swine fever virus [2,3]. Numerous studies have confirmed that PPV is associated with reproductive failures, including abortions, stillbirths, and mummified fetuses in sows, as well as a decline in semen quality in boars. These issues lead to substantial economic losses for the global pig farming industry [4,5]. Currently, PPV disease poses a significant challenge within the pig farming industry [6,7,8]. There is no specific treatment for PPV disease, and testing and vaccination are the main means of preventing and controlling the disease in clinical practice [9].

The advancements of genetic engineering technologies and new achievements in molecular biology research of parvovirus have introduced new research avenues for the development of PPV vaccines and detection methods. In this review, we concentrate on the research findings from the past decade and aim to summarize studies that have provided relevant insights into the biological characteristics of the virus as well as vaccine research.

## 2. Virological Characteristics of PPV

### 2.1. Virus Classification

PPV is a non-enveloped, single-stranded negative-sense DNA virus belonging to the family Parvoviridae and the genus Parvovirus. Parvoviruses are classified into Protoparvovirus (PPV1, PPV8), Tetraparvovirus (PPV2–PPV3), Copiparvovirus (PPV4–PPV6), or Chapparvovirus (PPV7) based on NS1 protein sequence homology [10], as shown in Table 1. The genus Parvovirus mainly includes Porcine Parvovirus (PPV), Canine Parvovirus (CPV), Goose Parvovirus (GPV), Feline Panleukopenia Virus (FPV), Human Parvovirus B19, etc., with highly genomic homology [11,12]. Eight genotypes of Porcine Parvovirus (PPV1–PPV8) exhibit distinct pathogenic profiles [6,10,13,14,15]. PPV1 was first reported in the 1960s and is considered the earliest PPV [6]. It is recognized as the primary etiological agent responsible for the porcine reproductive failure syndrome known as SMEDI (stillbirth, mummification, embryonic death, and infertility). So far, only PPV1 remains the only vaccine-targeted genotype to prevent reproductive failure in sows. Prevalent strains include virulent NADL-8 (PPV1c), attenuated NADL-2 (PPV1d), 27a-like (PPV1b), G1 (PPV1a), etc. Since 1855, PPV1 has evolved at a rate of 4.71 × 10^−5^ nucleotide substitutions per site per year. Most European strains belong to the PPV1a (G1) or PPV1b (G2 or 27a like) group, while Asian/American variants segregate into virulent PPV1c (such as NADL-8) or attenuated PPV1d (such as NADL-2) [16].

Novel genotypes (PPV2–PPV8), identified since 2001, demonstrate unresolved pathogenicity [17]. PPV2 was initially detected in Myanmar in 2001, from the lung tissue of pigs suffering from respiratory failure, which has led to its proposed role as a potential primary pathogen in the porcine respiratory disease complex. Additional studies have suggested PPV2 as a possible participant in porcine reproductive failure [18]. PPV3 was first identified in Hong Kong in 2010 from swine samples collected at slaughterhouses, and its pathogenicity is still unclear. PPV4 in 2010 and PPV5 in 2014 were both initially identified in the USA from porcine lung samples [15]. PPV6 is ubiquitous both in healthy and diseased pigs [19]. PPV7, first discovered in the United States in 2016 [20] and detected in multiple countries [21], shows high co-infection rates with porcine circovirus type 2 (PCV2) and ongoing mutation/recombination-driven pathogenicity risk warranting special attention. PPV8, the most recently reported PPV in 2022, was initially identified in the lungs of pigs with fever and respiratory signs in China [10,22,23]. The phylogenetic analysis of the novel PPV shows that the evolution of the PPV2 capsid is different from that of the original strain, and the nucleotide homology between the two different branches is low, ranging from 88% to 96% [24].

**Table 1:** Comparative analysis of the ITCV classification, molecular characteristics, and distribution of porcine parvovirus (PPV1 through PPV8).

Virus Types	ICTV Current Classification	Discovery Time	Genome Size (nt *)	ORF1 (aa #)	ORF2 (aa)	3′UTR	Global Distribution (Mainly Country) [15,17]	China Distribution (Mainly Province) [10,25]
PPV1	*Protoparvovirus ungulate1*	1965	5075	662	729	526	Pandemic disease worldwide	Henan, Shandong, Guangdong, Guangxi, Jiangxi Fujian, Jiangsu Sichuan, Chongqing, Hubei Hunan, Yunnan Heilongjiang, Jilin Liaoning, Tianjin Neimenggu
PPV2	*Tetraparvovirus ungulate3*	2001	5444	662	1032	135	Hungary, Romania, Germany, Poland, Europe, Korea, China, Japan, Vietnam, Thailand, USA, Mexico, South Africa	Henan, Shandong Guangdong, Guangxi, Jiangxi Fujian, Jiangsu, Sichuan, Chongqing Hubei, Hunan, Hebei, Yunnan, Heilongjiang, Shanxi, Tianjin, Anhui, Gansu
PPV3	*Tetraparvovirus ungulate2*	2008	5114	637	926	156	China, Thailand, Vietnam, Korea, Germany, Hungary Romania, Poland, South Africa, USA, Brazil, Mexico	Henan, Shandong Guangdong, Guangxi, Jiangxi Fujian, Jiangsu Sichuan, Chongqing, Hubei, Hunan, Hebei, Yunnan, Heilongjiang, Shangxi, Tianjin Anhui, Xijiang
PPV4	*Copiparvovirus ungulate2*	2010	5905	588	728	197	China, Korea, Thailand, Vietnam, Hungary, Romania, Germany, Poland, Europe, USA, Mexico, Brazil, South Africa	Henan, Shandong Guangdong, Guangxi, Jiangxi Sichuan, Chongqing, Hubei Hunan, Yunnan, Heilongjiang, Jilin Liaoning, Shanxi Gansu, Guizhou
PPV5	Not yet classified	2013	5805	601	991	9	USA, China, Korea, Poland, Mexico	Henan, Shandong, Guangdong, Guangxi, Sichuan, Chongqing, Hubei Yunnan, Heilongjiang
PPV6	*Copiparvovirus ungulate4*	2014	6148	662	1189	340	China, Korea, Poland, Russia, USA, Mexico	Henan, Shandong, Guangdong, Guangxi, Jiangxi, Sichaun, Chongqing, Hubei, Hunan, Hunan, Yunnan, Heilongjiang, Tianjin, Anhui
PPV7	*Chaphamaparvovirus ungulate1*	2015	4103	672	469	119	USA, China, Korea, Poland, Brazil, Colombia	Henan, Shandong, Guangdong, Guangxi, Sichaun, Chongqing, Hubei Hunan, Hunan, Yunnan, Heilongjiang, Tianjin, Jiangsu, Jiangxi, Gansu
PPV8	*Protoparvovirus ungulate4*	2022	4380	601	701	240	USA, China, Brazil Germany, Korea, Mozambique	Henan, Fujian

* nt: nucleotides; # aa: amino acids.

### 2.2. PPV Genomic

**Figure 1:**
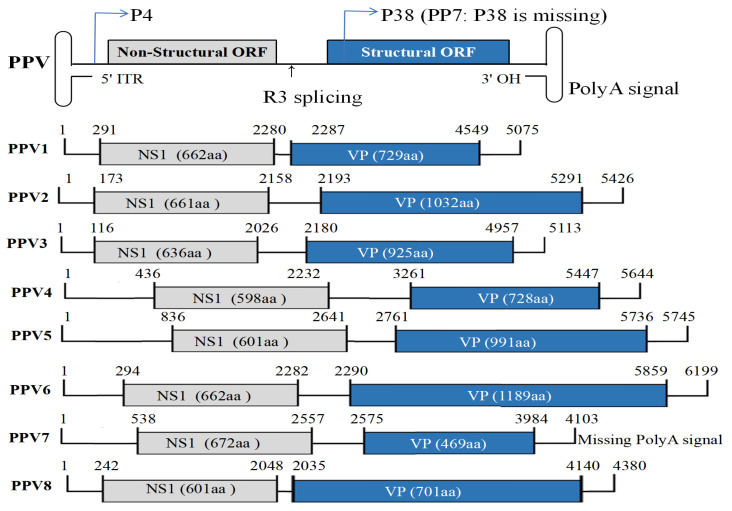
Genomic structures of porcine parvovirus with different genotypes. The arrow shown the alternative splicing region of the genome.

PPV is an autonomously replicating, single-stranded negative-sense DNA virus with a genome approximately 5000 base pairs (bp) in length [26,27]. As shown in Figure 1, the genome features palindromic sequences of 120 to 200 bp at both termini, forming a “Y”- or “T”-shaped hairpin structure at the 3′ end and a “U”-shaped hairpin structure at the 5′ end [28]. These hairpin structures play a crucial role in viral replication [29]. The PPV genome contains two promoters, P4 and P38, as well as two open reading frames (ORFs) [10]. Upon infection, the P4 promoter is initially activated to drive synthesis of R1 and R2 transcripts with a 5′ cap structure, yielding non-structural proteins NS1, NS2, and NS3 [30]. NS1 subsequently binds P38, driving R3 transcript expression and capping [31,32]. undergoes alternative splicing to encode structural proteins VP1/VP2 and SAT [33]. ORF1 of PPV encodes NS1 (84 kDa), which exhibits helicase activity through ATP binding/hydrolysis and regulates viral transcription via P38 binding, while NS2 (18 kDa) supports DNA replication [34]. The ORF1-encoded non-structural protein (NS1) exhibits high conservation across the Parvovirinae subfamily. As defined by the International Committee on Taxonomy of Viruses (ICTV), PPV strains are classified within the same genus when their NS1 amino acid sequences show over 85% identity [17]. The ORF2 encodes up to four structural proteins (VPs) forming the capsid [4].

### 2.3. PPV Virus Particle Structure and Structural Proteins

PPV is a non-enveloped virus (20–25 nm diameter) composed of viral capsid protein and genomic DNA [35]. The icosahedral capsid (T = 1 symmetry) comprises 60 identical copies of viral proteins. This assembly consists of approximately 90% VP2 and 10% VP1 [30], each containing eight antiparallel β-strands, one α-helix and four loops. Mature virions exhibit hexagonal symmetry with 5-fold, 3-fold, and 2-fold axes [5]. Around these axes, “spikes,” “canyon-like” structures, and “dimple-like” structures are formed, respectively. On the surface, a projection at the 3-fold axis, a depression or canyon around the 5-fold axis and a dimple on the 2-fold axis of symmetry can be observed. At the apex of the 5-fold axis, there is a channel, which serves as a pathway for viral gene exchange with the environment [36]. The 2- and 3-fold axes consist mainly of amino acids located in the subunit loops [37].

The PPV capsid comprises three structural proteins, VP1 (83 kDa), VP2 (64 kDa), and VP3 (60 kDa), all derived from ORF2 [4]. VP1 contains an additional 150 N-terminal residues harboring critical nuclear localization signals (Pat7: aa 3–9; bipartite NLS: aa 122–137) essential for early nuclear transport. Its conformational flexibility mediates viral entry. Meanwhile, VP2, generated by VP1 mRNA alternative splicing, mediates receptor recognition, adsorption, and entry. VP2 self-assembles into cytoplasmic trimers via a nuclear localization motif (K272/K275/K487/R576) and forms immunogenic capsids in the nucleus. Antigenically, both VP1 and VP2 harbor neutralizing epitopes. The N-terminal region of VP1 (RK R motif) induce neutralizing antibodies [38]. VP2 contains dominant linear (^89^ESGVAGQMV^97^) and conformational epitopes, with residues E89/S90/G91/V92/G94 critical for antibody binding [39]. VP2 alone has the ability to self-assemble into virus-like particles (VLPs), exhibits hemagglutinating activity, and can induce an immunoprotective response in the body [40]. N-terminal truncation (Δ1-47) of the VP2 preserves assembly, but deletion of Asn48 abolishes it. The N48K mutation further disrupts VLP stability, hemagglutination, and polymer formation [40]. Recombinant VP2-VLPs represent promising vaccine candidates due to their immunogenicity and biosafety. VP3 (60 kDa), arises from VP2 N-terminal cleavage (~25 aa), functions as a scaffold protein and also contributes to capsid formation and maintaining viral stability [4,36]. Together, these proteins orchestrate PPV infectivity, antigenicity, and structural integrity, making them key targets for vaccine development and antiviral strategies.

## 3. Epidemiology and Pathogenic Mechanism of PPV

### 3.1. PPV Epidemiology

PPV has the ability to infect all porcine species [18], with domestic pigs and wild boars as primary hosts, and primiparous sows are particularly susceptible. Transmission occurs via digestive/respiratory tracts, mating, placental transfer, and indirect routes (e.g., rodents, contaminated feed/water/utensils). The virus persists outside hosts for months and resists most disinfectants, facilitating widespread dissemination. Globally prevalent, PPV infects all breeds/ages, with infectious sources including feces, secretions, semen, carcasses, and contaminated materials. Viral shedding occurs via feces, secretions, and semen, with nucleic acid detectable in serum, liver, lungs, and lymphoid tissues. The main clinical symptoms of PPV infection include sow reproductive failure (abortion, fetal death, mummification) and piglet enteritis/respiratory lesions [41,42]; finishing pigs often show subclinical infections with transient lymphopenia (5–10 days post-infection) [9,22]. Fetal damage depends on gestational timing (≤70 days post-conception causes failure) [36,43] and strain virulence; low-pathogenic strains (e.g., NADL-2) rarely cross the placenta.

There are significant differences in pathogenicity among different subtypes of PPV. PPV1 was recognized as the primary etiological agent responsible for the porcine reproductive failure syndrome known as SMEDI (stillbirth, mummification, embryonic death, and infertility). Novel genotypes (PPV2–PPV8), identified since 2001, demonstrate unresolved pathogenicity [17]. The PPV2–4 were considered to be related to respiratory and intestinal diseases [24] (Lagan Tregaskis P, Staines A, Gordon A, Sheridan P, McMenamy M, Duffy C, Collins PJ, Mooney MH, Lemon K. Co-infection status of novel parvovirus’s (PPV2 to 4) with porcine circovirus 2 in porcine respiratory disease complex and porcine circovirus-associated disease from 1997 to 2012 [24].

PPV7, first discovered in the United States in 2016, shows a high recombination rate and high co-infection rate with PCV2. The ongoing mutation/recombination-driven pathogenicity risk and virulence warrant special attention. Co-infections with classical swine fever virus (SCFV) [44], porcine circovirus type 2 (PCV2) [24,45,46,47], porcine pseudorabies virus (PRV) [48,49,50], and porcine reproductive and respiratory syndrome virus (PRRSV) [51,52] are quite common.

### 3.2. Genetic Variation of PPV Genotypes

So far, eight confirmed PPV strains (PPV1 to PPV8) have been found in pig herds on all continents (except for PPV8, which is only found in Asia), with the highest prevalence of virus genomes detected in fattening and adult pig populations in pig farms [17], and the prevalence of different subtypes of PPV varies in different regions. At present, a total of eight serotypes (PPV1–PPV8) have been detected in China [53]. PPV2 is most common in China (40%) [18] and South Korean lung samples (32.6%) [51], while PPV3 dominates Colombia (40.1%) [54]. PPV7 was first discovered in the United States in 2016 [20] and has since been detected in China [14,21], Italy [8], America [55], and Europe.

PPV1–8 share high structural similarity but diverge genetically, with a mutation rate (10^−5^–10^−4^ substitutions/site/year) comparable to RNA viruses, predominantly in VP1/VP2 (affecting virulence/tropism/antigenicity) [17]. The VP1/VP2 genes serve as core markers for evolutionary analysis, with amino acid variations closely linked to geographic differentiation and host adaptation.

Phylogenetic analysis of VP2 genes (Supporting Information Figure S1) revealed four major genetic lineages: European (Group 1 + 2, e.g., Challenge, IDT vaccine); Asian (Group 3, e.g., Chinese/Korean strains, genetically similar to the NADL-2 vaccine strain); mixed lineage (Group 4, spanning continents, resembling Kresse); and an independent Asian clade. This grouping aligns with the “four-lineage” hypothesis proposed by Oh, W.T. et al. [11]. PPV1 has transcontinental distribution (Group 4, e.g., CN-JSZJ202104-1 clusters with Kresse, bootstrap = 100). PPV2 forms a robust Asian clade (e.g., PPV2-DSH1, bootstrap = 100). PPV3/4 are scattered, while PPV5-8 are predominantly Asian, consistent with independent Asian lineage differentiation [11].

Key divergent sites: Amino acid substitutions at positions 20, 82, 144, 215, 304, 378, 436, and 565 distinguish Asian and European strains (e.g., Thr20 in Asian VP2 vs. Ala20 in European VP2, Gly436 vs. Asp436, Ile565 vs. Val 565). These variations may affect viral replication, host tropism, and antigenicity. PPV1/2/5/7 in Asia cluster closely with vaccine strains (NADL-2/Kresse), while PPV3/4/6 show scattered distribution. Reversion to ancestral genotypes (e.g., Korean T142 = 1985 Kresse) and frequent PPV2 recombination (40% infection rate in China) drive evolution [11]. VP2 evolution (5.47 × 10^−5^ substitutions/site/year) and limited recombination suggest vertical inheritance. High genetic similarity between Asian strains and vaccines supports vaccine efficacy, but mixed lineages necessitate ongoing monitoring. No significant recombination events were detected [11].

### 3.3. Pathogenic Mechanism of PPV

The process of PPV infecting host cells involves precise multi-step regulation [36]. PPV initiates infection via sialic acid-mediated attachment, utilizing clathrin-dependent endocytosis (monomeric virions) or macropinocytosis (aggregated virions) [56]. Endosomal acidification (2–10 hpi) exposes the VP1 phospholipase A2 (PLA2) domain, enabling endosomal escape through membrane degradation [57]. Nuclear transport relies on temporal collaboration: microtubules mediate early (8–10 h) endosomal transport to the perinuclear region, while actin supports late (12–16 h) virion and protein nuclear localization.

The replication of PPV involves complex and diverse molecular mechanisms. The non-structural protein NS1 activates the NF-κB and the TLR9 signaling pathway, triggering the release of pro-inflammatory cytokines (IL-6) and inducing endoplasmic reticulum stress, thereby creating a permissive microenvironment [58]. When PPV infects pig fetal fibroblasts, autophagy is activated through the AMPK/Raptor/mTOR signaling pathway, promoting virus replication [59].

PPV infection alters cell death-associated genes and pathways [60]. In porcine placental trophoblasts cells (PTCs), viral DNA activates Z nucleic acid binding protein 1 (ZBP1) sensor, triggering necroptosis via the ZBP1/RIPK3/MLKL axis (independent of replication, linked to pathological damage) [61]. NS1/NS2 promote virion assembly by disrupting autophagic flux, while gallic acid in propolis inhibits replication by blocking caspase-dependent apoptosis [62].

### 3.4. Immune Regulatory Function of Structural Proteins

Viral persistence and transmission depend on immune evasion. In PPV-infected PK-15 cells, CD38 activates NLRP3 inflammasome by increasing reactive oxygen species (ROS) levels, and enhances TLR9/IFN—α/MX1 signaling, forming a pro-inflammatory and antiviral network; CD38 deficiency inhibits viral proliferation via SIRT1 upregulation, marking it as a therapeutic target [63]. PPV also activates autophagy in pig fetal fibroblasts via AMPK/Raptor to promote viral replication [64]. Host CpG methylation regulates viral gene expression, with Poly (rC) binding protein 1 (PCBP1) downmodulation enhancing infectivity (overexpression inhibits infection) [65]. PPV1–8 differ in CpG islands (PPV2/3: 12–16, PPV7: 6–11, others: 1–5) and GC content (PPV7 > 50%, others ≤ 50%) [66].

VP2, the major capsid protein, induces neutralizing antibodies via conformational epitopes, serving as a core subunit vaccine target [67,68]. Recent studies have revealed that the antigenic epitope diversity of VP2 may be associated with the immune escape characteristics of different PPV strains [40,69]. In summary, PPV pathogenicity involves (1) hijacking host metabolism (AMPK/mTOR) and cell death (necroptosis/autophagy) for replication; (2) evading immunity via epigenetic (CpG methylation) and pattern recognition receptor (CD38/TLR9) modulation; and (3) VP2-mediated immune response balance. These insights inform antiviral strategies targeting host factors or epigenetic regulators.

## 4. PPV Detection Technology

PPV-induced reproductive disorders in primiparous sows (asymptomatic estrus recurrence, mummified fetuses) pose a great threat to pig farms, seriously affecting the health and production performance of pig herds, and require rapid and accurate diagnosis. This article reviews the progress of detection technologies, covering detection methods based on pathogens, serology, molecules, and emerging platforms, but sensitivity and on-site applicability challenges still exist (Table 2).

### 4.1. Pathogen Detection

Pathogenic testing is the gold standard for diagnosing PPV infection, achieved through virus isolation, morphological identification, and observation of cytopathic effects (CPE)/hemagglutination assay (HA) validation. Although virus isolation has high specificity, its time-consuming nature, low sensitivity, and high operational requirements limit its use at the grassroots level. The specificity of CPA and HA detection is relatively poor, and a single method is difficult to distinguish pathogens with the same cytopathic characteristics and coagulation activity. It needs to be used in conjunction with other methods. Immunoelectron microscopy can directly observe virus particles at 20–22 nm, but it requires expensive equipment and professional operating techniques. In short, pathogen testing is mainly carried out by professional technicians in the laboratory.

### 4.2. Serological Detection

Serological testing indirectly diagnoses infections through antibody or antigen testing and is suitable for epidemiological investigations and immune efficacy evaluations. Serological detection techniques mainly include indirect immunofluorescence assay (IFA) [4], latex agglutination test (LAT) [74], hemagglutination inhibition test (HI) (OIE, 2021), virus neutralization test (VN) (Joo et al., 2000), enzyme-linked immunosorbent assay (ELISA) [76,77,78], and other methods. Among them, the hemagglutination inhibition test (HI) and enzyme-linked immunosorbent assay (ELISA) are easy to operate, do not require precise instruments and equipment, are convenient and timesaving, and are suitable for testing in breeding farms. Colloidal gold immunochromatography based on antigen–antibody reaction are point-of-care testing (POCT) methods. It has high convenience and can be used for field and field testing, although this method has not been included in national standards in China.

### 4.3. Molecular Biology Diagnostic Techniques

Molecular diagnostic technology, with nucleic acid amplification as its core, breaks through the sensitivity and timeliness bottlenecks of traditional methods. Methods including nucleic acid probe technology, polymerase chain reaction, real-time fluorescence quantitative PCR (qPCR) detection methods are some of the main methods for clinical testing.

In the field of pig disease diagnosis, multiple joint inspection technology has become a key breakthrough in improving efficiency. Multiplex qPCR can simultaneously detect mixed infections of multiple viruses significantly improving detection efficiency [48,87]; recombinant enzyme polymerase amplification (RPA), combined with the CRISPR/Cas12a system, RPA-CRISPR/Cas12a [88] and a combination of enzyme catalyzed recombinase amplification (ERA)-CRISPR/Cas12a system and lateral flow test strip (LFT) has shown 30 min on-site detection (3.75 × 10^2^ copies/μL limit) [89].

Gene chips (34.5 ng/μL, 100% consistency with PCR) [91], a non-radioactive slit hybridization method using digoxin labeled DNA probes (1ng DNA, or 100PFU) or biotinylated RNA probes (0.1 ng DNA, or 10PFU) [82], microfluidic LAMP chips (10^1^ copies/μL for PPV2) [92], and biosensors show promise but face cost/stability barriers.

The detection of PPV virus is an important technical means for comprehensive virus prevention and purification. The above detection methods are based on different technical paradigms and meet the needs of different application scenarios. The pathogen isolation method is accurate and reliable, but the operation is cumbersome and time-consuming; The serological detection method is relatively convenient, but it has cross reactivity and relies on fresh red blood cells, which is not conducive to on-site operation. Molecular technology is fast and convenient, but it requires equipment and specialized laboratories. In the future, portable CRISPR/nanomaterial POCT and recombinant enzyme polymerase amplification (RPA) multiplex detection can be used for on-site testing. In the future, research on artificial intelligence-assisted result reporting and epidemic prediction, fully automated microfluidic chips, and high-sensitivity biosensors will become the development direction of PPV detection technology.

## 5. PPV Vaccines

Vaccination is the main method for preventing and controlling PPV in livestock farms. Due to high biosafety risks and a short immune protection period, PPV attenuated live vaccines have been gradually phased out. At present, the vaccines mainly used in clinical practice are inactivated vaccines, subunit vaccines, and virus-like particle (VLP) vaccines, while viral vector vaccines and nucleic acid vaccines represent emerging technological directions. In this article, we reviewed the application of various vaccine technology platforms in the development of PPV vaccines (Table 3), and summarized the PPV vaccines that have been launched in China (Table 4).

### 5.1. Inactivated Vaccines

The application of inactivated PPV vaccines for disease prevention was first documented in 1977 [115]. Developed via chemical inactivation (formaldehyde, β-propiolactone, N-acetyl ethylenimine (AEI)) of PPV strains with adjuvants, these induce robust antibodies and exhibit good stability but fail to prevent viral shedding. Adjuvant advancements (e.g., N-2-hydroxypropyl trimethyl ammonium chloride chitosan (N-2-HACC) [93], ophiopogon polysaccharide liposome (OPL) [95], propolis [116]) enhance immunogenicity, with propolis outperforming oil-emulsion/aluminum salt adjuvants in early antibody production. In addition, molecular adjuvants such as transfer factors and bacterial flagellar proteins have also been used to enhance the immune efficacy of inactivated vaccines. Transfer factor (TF) enhances cellular immune response but does not affect antibody titers [117].

### 5.2. Attenuated Vaccines

PPV attenuated vaccines refer to vaccines prepared using non-pathogenic PPV attenuated strains or virulent strains that have been attenuated, like NADL-2 strain (54 passages) [99]. Attenuated vaccines offer efficient immune protection but pose reversion risks and storage challenges [118]. Replaced by inactivated vaccines, PPV attenuated vaccines are no longer used in China.

### 5.3. Viral Live-Vectored Vaccines

Viral live-vectored vaccines are engineered by integrating the PPV antigen gene (e.g., VP2) into replication-competent viral vectors. Following host cell infection, these vectors express the heterologous antigen, thereby eliciting robust humoral and cellular immunity. Engineered by inserting PPV VP2 into vectors (e.g., Ad5, PRV), these elicit humoral/cellular immunity but face pre-existing vector immunity and payload limits. Examples include Adenovirus 5 (Ad5)-vectored VP2 (Ad5-VP2) [119] and trivalent PRV-vectored vaccines targeting PRV/FMDV/PPV, highlighting multivalent potential [101]. These findings highlight the platform’s potential for multivalent vaccine development, contingent on resolving vector-specific immune interference and payload constraints.

### 5.4. Recombinant Subunit Vaccines

Recombinant subunit vaccines mainly express PPV key antigens like VP2 protein through genetic engineering methods, purify them and mix them with adjuvants. The PPV subunit vaccine can induce high titers of neutralizing antibodies and has high biological safety. For example, carbomer-adjuvanted (ReproCyc) ^®^ VP2 vaccines prevent viremia and protect fetuses for ≥6 months [103]; N-2-hydroxypropyl trimethylammonium chloride chitosan (N-2-HACC)-adjuvanted VP2 induces long-term protection (100% efficacy) [104].

### 5.5. PPV VLP Vaccines

Self-assembled from VP2, these empty capsids lack viral nucleic acid, inducing high neutralizing antibodies and safety. Research shows that prokaryotic/eukaryotic-expressed VLPs (*Escherichia coli* (*E. coli*), Nicotiana benthamiana, Bac-to-Bac system) protect sows against heterologous PPV strains with complete fetal protection. For example, VLPs vaccine from *E. coli* expressed VP2 protein immunization, resulting in complete fetal protection against PPV infection in primiparous sows [68,108,120]; other VLP vaccines, from Kluyveromyces marxianus [121], Nicotiana benthamiana [109] and Bac-to-Bac expresssion system [67], induce long-term and complete fetal protection. Six VLP vaccines have entered clinical trials in China. VLPs outperform inactivated vaccines in cellular immunity, with stable batch consistency [106].

### 5.6. Nucleic Acid Vaccines

Nucleic acid vaccines, delivering antigen-encoding DNA/RNA via host expression systems, show promise for PPV control. Immunization of mice with pcDNA-VP2, a plasmid encoding the PPV VP2 protein, effectively elicits both humoral and cellular immune responses [122]. Multi-epitope constructs induce dual PCV/PPV immunity in mice [123], with plasmids like pCI-VP2.ORF2B and IL-2/IFN-γ-adjuvanted vectors boosting humoral/cellular responses [110,111,112,113,114]. However, research on these nucleic acid vaccines lacks immune protection trials targeting pigs. Compared to mice, it is more difficult to induce high titer immune protection in pigs. Adopting more efficient nucleic acid delivery strategies, such as using nano molecular materials as delivery media or introducing more effective molecular adjuvants, such as bacterial flagellin and granulocyte colony-stimulating factor (GM-CSF), may be an effective direction to solve this problem.

So far, a total of 10 vaccines against porcine parvovirus disease have been approved for marketing in China, including 7 virus inactivated vaccines and 3 virus vector inactivated vaccines (Table 4).

## 6. Conclusions and Future Perspectives

### 6.1. Key Conclusions

This comprehensive review synthesizes the current understanding of porcine parvovirus (PPV) in China, highlighting its significant economic impact on intensive pig production due to reproductive failures and evolving viral epidemiology. PPV exhibits remarkable genetic diversity, with eight confirmed genotypes (PPV1–PPV8) circulating globally. PPV1 remains the primary vaccine target, but emerging genotypes (PPV2–PPV8) are increasingly prevalent in Chinese herds, particularly PPV2. Continuous evolution, driven by nucleotide substitutions (e.g., VP2 at ~5.47 × 10^−5^ subs/site/year) and recombination (notably in PPV2), poses challenges for diagnostics and vaccine matching. Critical amino acid variations in structural proteins (e.g., VP2 residues 20, 82, 436, 565) underpin geographic adaptation and potential antigenic drift.

PPV infection dynamics are complex, influenced by host factors (e.g., primiparous sow susceptibility, gestational timing ≤70 days), co-infections (notably PCV2, PRRSV, PRV, CSFV), and environmental persistence of the non-enveloped virion. Novel genotypes show distinct tissue tropisms and associations (e.g., PPV2–PPV4 with respiratory/intestinal issues, PPV7 co-infection with PCV2), though their individual pathogenic contributions require further elucidation.

PPV pathogenesis involves intricate virus–host interactions: sialic acid-mediated entry, endosomal escape via VP1 PLA2, NS1-mediated immune modulation (NF-κB/TLR9 activation, ER stress), and exploitation of host pathways (AMPK/mTOR autophagy, ZBP1/RIPK3/MLKL necroptosis). Immune evasion strategies include modulation of CD38/NLRP3/TLR9 signaling, autophagy manipulation, and epigenetic regulation via CpG methylation (varying significantly between genotypes).

While molecular techniques (multiplex qPCR, CRISPR-based platforms like RPA-CRISPR/LFT) offer high sensitivity and speed for detecting PPV and co-infections, significant gaps remain in affordable, point-of-care (POCT) tools suitable for grassroots veterinary use. Standardization and validation across diverse PPV genotypes are critical needs.

Current PPV vaccines, predominantly inactivated whole-virus or subunit/VLP targeting PPV1 VP2, effectively control homologous PPV1-induced reproductive failure. However, they exhibit limited cross-protective efficacy against heterologous strains (especially emerging genotypes) and fail to prevent viral shedding. While VLPs show superior immunogenicity and safety, and represent the most promising next-generation platform, challenges in achieving broad-spectrum protection, thermostability, and cost-effective large-scale production persist. Viral-vectored and nucleic acid vaccines offer theoretical advantages but lag in practical efficacy and stability.

### 6.2. Future Perspectives

Addressing the persistent burden of PPV in China and globally requires a multifaceted, forward-looking approach. Nationwide, real-time PPV genotype and strain monitoring networks integrating next-generation sequencing (NGS) should be established. Mapping the prevalence, virulence determinants, and evolutionary trajectories of PPV2–PPV8, particularly their role in co-infections and recombination events, should be emphasized. Rational design of multivalent VLP vaccines should be prioritized, incorporating conserved, immunodominant epitopes from prevalent genotypes (especially PPV1, PPV2, PPV7), such as conserved epitopes “ESCVACQMV” loop from VP2 protein. Structural biology (cryo-EM) and computational epitope prediction should be utilized to engineer chimeric VLPs with broad cross-neutralizing capacity. Promising technologies (CRISPR-Cas systems, microfluidics, nanomaterials) should be translated into robust, affordable, and user-friendly POCT devices for on-farm, multiplex detection of PPV genotypes and major co-pathogens. AI should be integrated for automated result interpretation and epidemiological data analysis. The isothermal amplification PCR technology combined with visualization technology may be an important method for future grassroots veterinary testing. Closer collaboration should be fostered between virologists, immunologists, structural biologists, bioinformaticians, and veterinary practitioners. Field-validation of new vaccines and diagnostics should be prioritized, ensuring solutions are tailored to the specific epidemiological and operational challenges of Chinese pig production systems.

In conclusion, while significant strides have been made in understanding PPV virology and control, the evolving landscape demands a shift from reactive measures to proactive, precision-based strategies. Leveraging multi-omics data, structural insights, and innovative biotechnology holds the key to developing next-generation vaccines with broad-spectrum efficacy and point-of-care diagnostics, ultimately bridging the gap between basic science and effective PPV management for sustainable swine production.

## Figures and Tables

**Table 2:** Serological detection techniques.

Method		Target	Sensitivity	Utility	Ref.
Pathogen detection	Virus isolation (CPE)	Virus	--	Discovery of new strains; Analysis of virulence genes; Research and traceability	[70]
	HA	Antigen	10^3^ copies/μL		[71]
	IFA	Antigen	10^2^ copies/μL		[4,72]
	Immunoelectron microscopy	virions	10^2^ copies/μL		[73]
Serological detection techniques	IFA	Antigen	10^2^ copies/μL	Tissue localization	[4,71]
	LAT	IgM antibodies	10^2^ copies/μL	Applicable for qualitative IgM antibody screening, suitable for on-site initial screening	[74]
	* HI	Antibody	10^3^ copies/μL	OIE-standardized monitoring	[75]
	ELISA	Antibody Antigen	10^2^ copies/μL	High-throughput screening	[76,77,78]
	VN	Neutralizing Ab	10^2^ copies/μL	Gold standard	[79]
	Dot-PPA-ELISA	Antibody Antigen	10^3^ copies/μL		[80]
	Immunochromatographic strip	Antibody	10^2^ copies/μL	Realize rapid on-site detection within 15 min	
Molecular biology diagnostic techniques	Nucleic acid probe	nucleic acid	DNA probes: 10^2^ copies/μL RNA probes: 10^2^ copies/μL	Population screening; Epidemiological investigation	[81]
					[82]
	PCR	nucleic acid	10^2^ copies/μL		[83]
	nest-PCR	nucleic acid	10 copies/μL		[84]
	LAMP	nucleic acid	10 copies/μL		[85]
	qPCR	nucleic acid	10 copies/μL		[23,43]
	multiplex PCR	nucleic acid	10 copies/μL	For co-infections with PCV2/PRV	[44,54,86]
	Multiplex qPCR	nucleic acid	10 copies/μL	Rapid identification of PPV and other pathogens (such as PCV2, CSFV)	[48,87]
Emerging detection technologies	CRISPR-RPA-LFT	nucleic acid	100 copies/μL	POCT technology innovation; Grassroots on-site testing	[88]
	CRISPR-ERA-LFT	nucleic acid	10^2^ copies/μL		[89]
	SECGA	Antigen	10^2^ copies/μL	Grassroots on-site testing	[90]
	Gene chips	nucleic acid	10^2^ copies/μL	100% consistency with PCR	[91]
	Next genome sequencing (NGS)	nucleic acid	10^2^ copies/μL	Discovery of new strains; Analysis of virulence genes; Transcriptome screening for novel biomarkers	[54]

* HI detects antibodies at day 5 post-infection, peaking at days 12–14; LAT provides IgM screening in 10 min but lacks quantitation. Abbreviations: latex agglutination test (LAT); hemagglutination (HA); hemagglutination inhibition test (HI); indirect immunofluorescence assay (IFA); virus neutralization test (VN); enzyme-linked immunosorbent assay (ELISA); loop-mediated isothermal amplification (LAMP); silver enhanced colloidal gold technology (SECGA); recombinant enzyme polymerase amplification (RPA), combined with the CRISPR/Cas12a system (CRISPR-RPA); enzyme catalyzed recombinase amplification (ERA)-CRISPR/Cas12a system (CRISPR-ERA); lateral flow test strip (LFT); next genome sequencing (NGS); plaque-forming units (PFU); porcine parvovirus (PPV), porcine circovirus type 2 (PCV2), pseudorabies virus (PRV), and swine fever virus (CSFV).

**Table 3:** Overview of vaccine development for PPV diseases.

Platform	Patterns	Cross-Protection	Development Time	Cross-Protection	Field Stability	Ref.
Inactivated (Chemical or physical methods)	Formaldehyde inactivation PPV NADL-2	Titer of neutralizing antibodies in pregnant sows ≥ 1:64; Protection rate against fetal vertical infection > 90%;	6–8 months	Moderate	Excellent	[16,67,93,94,95,96]
	Adjuvant: CpG-ODN	The cytokine (IFN—γ) level increased by 2 times, and the antibody affinity was enhanced				
	Inactivated PPV + Porcine Circovirus Type 2 (PCV2) bivalent vaccine	The protection rates for PPV and PCV2 are 92% and 88%, respectively				
Attenuated vaccine	PPV NADL-2	Reduce virus virulence, preserve replication ability and immunogenicity through continuous passage or genetic modification	12 months or langer	Moderate	Good	[97,98,99]
Viral live-vector vaccine	Adenovirus 5 Ad5-VP2	After virus attack, the viral load decreased by 80–85% in mice;	6–8 months	Moderate	Thermal stability, multivalent potential	[100,101]
	PRV-vectored trivalents	Vector-delivered VP2 expression humoral/cellular immunity;				
	SPV-VP2	The fetal protection rate of sows after immunization is greater than 95%, and there is no pre-existing immune interference against porcine pox virus vectors				
Subunit	(Bac-to-Bac) VP2	After immunization with sows, the neutralizing antibody titer is ≥1:128, and the vertical transmission protection rate is >90%;	3–6 months	Limited	Good	[67,102]
	VP2/Carbomer (ParvoFLEX)	ReproCyc^®^ Prevents viremia; 6-month heterologous protection				[103]
	VP2/N-2-HACC	Water-soluble chitosan 100% protection; HI titers;				[104]
	VP2/*E. coli*	The antibody titer is lower than that of the insect cell system and requires the use of Freund’s adjuvant, with a protection rate of about 80%				[40,105]
VLP	(Bac-to-Bac) VP2 (VLP)	The titer of neutralizing antibodies is three times higher than that of inactivated vaccines	6–8 months	High	Moderate	[106,107]
	*E. coli*: Endotoxin-free VLPs	(HA = 2^19^; Shen et al., 2024)				[68,108]
	*Nicotiana benthamiana* VP2 (VLP)					[109]
Nucleic acid *	PPV VP2 DNA + IL-2	The antibody response of the mouse model is good, and the protection rate in pig experiments is only the same as that of inactivated vaccines 60–70%	2–4 months	Theoretical	Poor (LNPs require −80 °C)	[110,111,112,113,114]
	PEI-nanoparticles encapsulate VP2 plasmid	Improve transfection efficiency, 2-fold increase in antibody titers and protection rate in pig experiments is 75%				

* Future priorities: Multivalent formulations (e.g., PPV-PCV2-PRRSV) and thermostable delivery systems.

**Table 4:** Available commercial vaccines against PPV in China.

	Name	Strain	Vaccine Type	Main Research Institutions	Release Time
1	Porcine parvovirus baculovirus vector inactivated vaccine (Strain rPP03)	rPP03	inactivated virus vector vaccine	Yangzhou Youbang Biopharmaceutical Co., Ltd. Hangzhou, China	2025/8
2	Porcine parvovirus baculovirus vector inactivated vaccine (Strain HP-SC-VP2)	HP-SC-VP2	inactivated virus vector vaccine	Huapai Biotechnology (Group) Co., Ltd. Chengdu, China	2025/7
3	Porcine parvovirus baculovirus vector inactivated vaccine (PPV-VP2)	--	inactivated virus vector vaccine	Pulike Bioengineering Co., Ltd. Luoyang, China	2023/6
4	Porcine parvovirus disease inactivated vaccine (Strain SC1)	SC1	inactivated vaccine	Huapai Biotechnology (Group) Co., Ltd. Chengdu, China	2020/10
5	Porcine parvovirus disease inactivated vaccine (Strain CG-05)	CG-05	inactivated vaccine	Guangdong Wenshi Dahua Nong Biotechnology Co., Ltd. Guangzhou, China	2019/6
6	Porcine parvovirus disease inactivated vaccine (Strain NJ)	NJ	inactivated vaccine	National Engineering Technology Research Center for Animal Biological Products Luoyang, China	2016/07
7	Porcine parvovirus disease inactivated vaccine (Strain BJ-2)	BJ-2	inactivated vaccine	Yangzhou Youbang Biopharmaceutical Co., Ltd. Yangzhou, China	2012/03
8	Porcine parvovirus disease inactivated vaccine (Strain YBF01)	YBF01	inactivated vaccine	Qingdao Yibang Bioengineering Co., Ltd. Qingdao, China	2011/08
9	Porcine parvovirus disease inactivated vaccine (Strain L)	L	inactivated vaccine	Harbin Pharmaceutical Group Biological Vaccine Co., Ltd. Harbin, China	2010/09
10	Porcine parvovirus disease inactivated vaccine (Strain WH-1)	WH-1	inactivated vaccine (Suspension cultured cells)	Huazhong Agricultural University Wuhan, China	2024/5/7 (Production process change)

## Supplementary Materials

The following supporting information can be downloaded at: https://www.mdpi.com/article/10.3390/v17091262/s1, Figure S1: Phylogenic trees created with the amino acid of major structural protein (VP2) sequences.
