# Supplementary material for: Porcine Parvovirus in China: Recent Advances, Epidemiology, and Vaccine Strategies
Source: Viruses. 2025 Sep 18;17(9):1262. doi: 10.3390/v17091262 (PMC12474210; doi:10.3390/v17091262)
Supplement: Supplementary file 1 [file viruses-17-01262-s001.zip › viruses-3833171-supplementary.pdf]

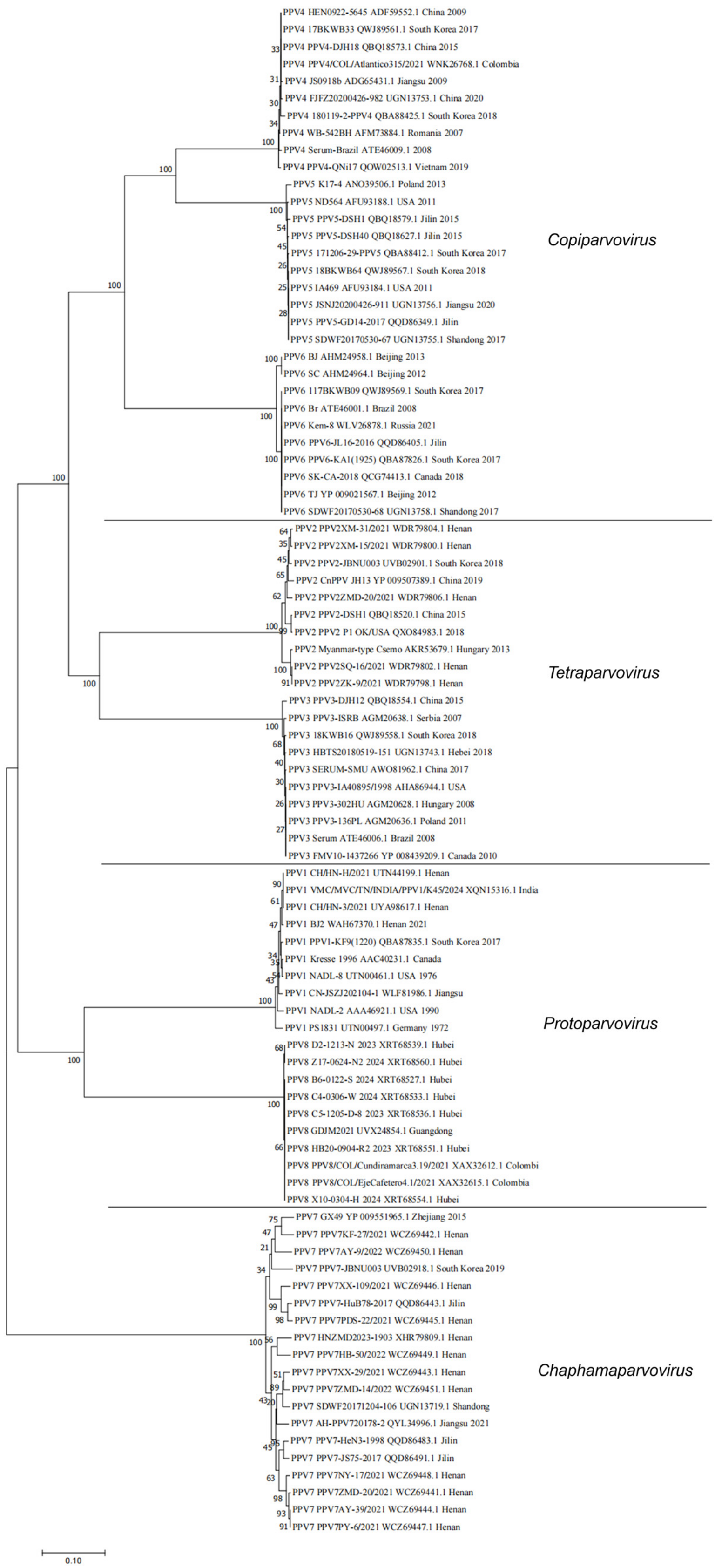

Supporting information Figure S1 Phylogenic trees created with the amino acid of major structural protein (VP2) sequences.
